# Membrane progesterone receptor α (mPRα) enhances hypoxia-induced vascular endothelial growth factor secretion and angiogenesis in lung adenocarcinoma through STAT3 signaling

**DOI:** 10.1186/s12967-022-03270-5

**Published:** 2022-02-05

**Authors:** Zhi Xia, Jian Xiao, Ziyu Dai, Qiong Chen

**Affiliations:** 1 Department of Geriatrics, Respiratory Medicine, Xiangya Hospital, Central South University, Changsha, Hunan China; 2 National Clinical Research Center for Geriatric Disorders, Xiangya Hospital, Central South University, Changsha, China

**Keywords:** Lung adenocarcinoma, Membrane progesterone receptor α (mPRα), Vascular endothelial growth factor (VEGF), Hypoxia, Angiogenesis, STAT3 signaling

## Abstract

Lung cancer remains a huge challenge to public health because of its high incidence and mortality, and lung adenocarcinoma (LUAD) is the main subtype of lung cancer. Hypoxia-induced vascular endothelial growth factor (VEGF) release and angiogenesis have been regarded as critical events in LUAD carcinogenesis. In the present study, membrane progesterone receptor α (mPRα) is deregulated within LUAD tissue samples; increased mPRα contributes to a higher microvessel density (MVD) in LUAD tissues. mPRα knockdown in A549 and PC-9 cells significantly inhibited STAT3 phosphorylation, as well as HIF1α and VEGF protein levels, decreasing cancer cell migration and invasion. The in vivo xenograft model further confirmed that mPRα enhanced the aggressiveness of LUAD cells. Furthermore, mPRα knockdown significantly inhibited hypoxia-induced upregulation in HIF1α and VEGF levels, as well as LUAD cell migration and invasion. Under the hypoxic condition, conditioned medium (CM) derived from mPRα knockdown A549 cells, namely si-mPRα-CM, significantly inhibited HUVEC migration and tube formation and decreased VEGF level in the culture medium. In contrast, CM derived from mPRα-overexpressing A549 cells, namely mPRα-CM, further enhanced HUVEC migration and tube formation and increased VEGF level under hypoxia, which was partially reversed by STAT3 inhibitor Stattic. In conclusion, in LUAD cells, highly expressed mPRα enhances the activation of cAMP/JAK/STAT3 signaling and increases HIF1α-induced VEGF secretion into the tumor microenvironment, promoting HUVEC migration and tube formation under hypoxia.

**Supplementary Information:**

The online version contains supplementary material available at 10.1186/s12967-022-03270-5.

## Introduction

Lung cancer is one of the most commonly seen cancers with the second-highest incidence rate among men and women globally. Lung cancer mortality ranks first among all cancers [1–3]. NSCLC (non-small cell lung cancer) accounts for approximately 85% of lung cancers, and LUAD (lung adenocarcinoma) is the most common subtype of NSCLC [2]. As the high-throughput sequencing technique develops, gene deregulation in LUAD has been gradually identified. Thus, further investigating the specific role of these deregulated genes in LUAD might provide an in-depth understanding of the occurrence, development, and treatment of LUAD.

Unlike lung squamous cell carcinoma, which is prone to occur in men and is closely related to smoking [4], LUAD is often in women who do not smoke [5, 6]. Even with LUAD, male and female patients have significant differences in treatment response and prognosis [7, 8]. Thus, it is reasonable to hypothesize that the onset and development of LUAD might be related to female sex hormones, mainly including estrogen and progesterone. Previous studies indicate that the receptors of estrogen, ERα (estrogen receptor α) and ERβ (estrogen receptor β), appear to be expressed within pulmonary cancer/LUAD, and both can mediate the oncogenic role of estrogen in lung cancer or act as oncogenic factors themselves [9–11]. As for progesterone, in addition to the canonical progesterone receptor (PR), membrane progesterone receptors (mPRs) attract more and more attention because of their potential role in cancers. One of the mPRs, mPRα, is expressed within various types of cancer cells, such as breast carcinoma cells [12], ovarian cancer cells [13], astrocytoma cells [14], and glioblastoma cells [15]. mPRα has been reported to promote the development of breast cancer [12, 16, 17]. Although mPRα expression is upregulated in LUAD tissues, according to data from GEPIA (Gene Expression Profiling Interactive Analysis), the independent role of mPRα in LUAD remains unclear.

Angiogenesis, the sprouting of capillaries from preexisting blood vessels, is crucial for solid tumor cell growth, invasion, and metastasis [18, 19]. In an oxygen-deficient environment, the hypoxia‐inducible factor (HIF1α) is a key transcriptional mediator of the response to hypoxic conditions. HIF1α regulates the secretion of several angiogenic factors, including VEGF (vascular endothelial growth factor), from tumor cells into cancer microenvironment [20], subsequently promoting angiogenesis. Moreover, several signaling pathways are involved in tumor neovascularization. For example, exogenous IGF-1 leads to the activation of IGF1R and STAT3 and upregulates the protein contents of HIF1α and the transcription activity of HIF1, resulting in VEGF release and angiogenesis [21]. CXCR4 enhances tumor blood vessel formation within gastric carcinoma through JAK2/STAT3 activation [22]. Notably, increased activity of STAT3 enhances the activity of HIF1α, and STAT3 is another factor able to bind to HIF1α promoter within transformed cell lines and growing tumors [23]. However, whether mPRα is involved in the JAK/STAT3 signaling-induced activation of HIF1α/VEGF in LUAD is unclear.

Progesterone signaling initiated at the plasma membrane via G protein activation in numerous target cells is mediated by mPRα [24–28]. A G protein-binding domain has been partially characterized on the third intracellular loop of mPRα at a similar position to that shown to be important for G protein signaling in G protein-coupled receptors [28]. Progesterone has been shown to activate G protein via mPRs within many vertebrate cellular types [24, 28–31]. The fish mPRα is coupled to an inhibitory G protein, thereby mediating progesterone-dependent MAPK activation and inhibiting cAMP production [32]. In the lactotroph population, mPRα can mediate the suppressive role of progesterone in the secretion of PRL via reduced cAMP levels and decreased TGFβ1 activation within the lactotroph population [33]. Thus, it's reasonable to hypothesize that mPRα might affect hypoxia-induced angiogenesis in LUAD through cAMP/JAK/STAT3 signaling and HIF1α-induced VEGF release.

The effects of mPRα on cancer cells’ capacity to proliferate, migrate, and invade has been reported within glioblastoma cells [15]. Herein, mPRα expression and microvessel density (MVD) within LUAD tissue samples were evaluated. mPRα knockdown or overexpression was generated in LUAD cells, and the roles of mPRα in cAMP concentrations, the protein levels of STAT3 signaling factors, HIF1α, and VEGF, and LUAD cell migration, invasion in vitro and tumor growth in vivo were investigated. As for hypoxia-induced angiogenesis, firstly, we exposed LUAD cells to a normoxic or hypoxic environment and examined the effects of mPRα knockdown on HIF1α and VEGF protein levels, LUAD cell migration and invasion; secondly, culture medium derived from mPRα knockdown or overexpressing LUAD cells was used for HUVEC (human umbilical vein endothelial cell) culture, and the cumulative tube length and cell migration ability of HUVECs were examined. In summary, we attempt to validate the specific effect and potential mechanism of mPRα for hypoxia-induced angiogenesis in LUAD.

## Materials and methods

### Clinical sampling

A total of 20 paired lung adenocarcinoma (LUAD) tissues and adjacent noncancerous tissues were obtained from patients diagnosed with LUAD and underwent surgical resection at Xiangya Hospital of Central South University with the informed consent signed. All samples were stored at – 80 ℃ or formalin-fixed and paraffin-embedded. All of the experimental procedures were performed under the Ethics Committee of Xiangya Hospital of Central South University (No.202009803).

### Cell lines

Lung adenocarcinoma cell line PC-9 was obtained from Merck (90,071,810; Kenilworth, NJ, USA) and cultured in RPMI 1640 plus 2 mM Glutamine and 10% Fetal Bovine Serum (FBS; Invitrogen, Carlsbad, CA, USA). Lung adenocarcinoma cell line A549 (adenocarcinomic human alveolar basal epithelial cell line) was procured from ATCC (CCL-185; Manassas, VA, USA) and cultured in F-12 K Medium (30–2004; ATCC) plus 10% FBS. HUVEC was procured from ATCC (PCS-100–010) and cultured in EGM™-2 Endothelial Cell Growth Medium (CC-3162; Lonza, Basel, Switzerland). All the cells were cultured at 37 ℃ in 5% CO_2_.

### Cell transfection and treatment

mPRα knockdown was generated in target cells by transducing mPRαt arget-specific siRNA (si-mPRα) synthesized by GenePharma (Shanghai, China). Scramble siRNAs were used as a negative control (si-NC). mPRα overexpression was generated by the transfection of mPRα-overexpressing vector (mPRα) synthesized by GenePharma. The empty vector was used as a negative control (NC). Following the protocols, si-mPRα/si-NC or mPRα/NC was transfected into LUAD cells (A549 and PC9), respectively, using lipofectamine 3000 (Invitrogen). The sequence of siRNA was listed in Additional file 2: Table S1.

For STAT3 inhibition, cells were treated with Stattic (5 μM) for 24 h. For hypoxia treatment, transfected LUAD cells and HUVECs were exposed to 1% O_2_ for 24 h in a tri-gas incubator (SANYO, Japan).

### Polymerase chain reaction (PCR)-based analyses

Total RNA was extracted, processed, and examined for the expression of target mRNA following the methods described previously [34]. mRNA expression was detected by SYBR green PCR Master Mix (Qiagen, Hilden, Germany) using β-actin (for mRNA examination) as an endogenous control. The data were processed using a 2^−ΔΔCT^ method. The primers are listed in Additional file 2: Table S1.

### Immunohistochemical (IHC) staining

Clinical and mice tumor tissue samples were sectioned into 5 μm-thick slices, deparaffinized in xylene, and rehydrated in a series of graded alcohols. The antigen retrieval was performed by immersing the slides in sodium citrate. The endogenous peroxidase was blocked by a 10-min incubation with 3% H_2_O_2_. Next, the slices were incubated with primary antibody anti-mPRα (ab75508, Abcam, Cambridge, UK), anti-CD31 (11265–1-AP, Proteintech, Wuhan, China), ki-67 (27309–1-AP, Proteintech), or PCNA (10205–2-AP, Proteintech) overnight at 4 °C, washed thrice by PBS, and incubated with horse-radish peroxidase (HRP)-conjugated secondary antibody (ab205718, Abcam) for 30 min. Finally, the immunostaining was performed using a DAB (diaminobenzidine) Substrate Kit (ab64238; Abcam).

### Evaluation of microvessel density

A total of 10 LUAD tissue samples were used for MVD assessment, 5 with relatively high mPRαexpression and 5 with relatively low mPRα expression. Each section was examined at low magnification (40 ×), the area with the highest vascular density (hot spot) was selected, and then observed at high magnification (100 ×). CD31-positive vessels were counted in three to five hot spots, respectively. Only continuous, membranous staining was considered as positive. Any large microvessel with a lumen or any single, separated endothelial cell was given a count of one. The vessels were counted within the epithelium and at the epithelium/stroma edge.

### Immunoblotting

Total protein was extracted, resolved on 10% SDS-PAGE, and transferred onto polyvinylidene fluoride (PVDF) membranes. Non-specific bindings were blocked by incubation with 5% nonfat dry milk in Tris-buffered saline Tween (TBST) for 2 h. After that, the membranes were probed with appropriate primary antibodies at 4 °C overnight, followed by another incubation with the corresponding secondary antibodies for 2 h at room temperature. The primary antibodies used are as follows: anti-mPRα (ab75508, Abcam), anti-STAT3 (10,253-2-AP; Proteintech, Wuhan, China), anti-p-STAT3 (ab76315, Abcam), anti-VEGF (66,828-1-Ig, Proteintech), anti-HIF1α (20,960-1-AP, Proteintech), and β-actin (endogenous control; 6008-1-Ig, Proteintech).

### Migration and invasion determined by Transwell assays

For invasion determination, cells, 48 h after transfection, in the logarithmic growth phase were plated in serum-free media with mitomycin C (1 μg/ml, Sigma) and added to each of the Matrigel-containing 8 µm Transwell upper-chamber. In the meantime, the bottom chambers were added with a medium containing 10% FBS as a chemoattractant. After a 48-h incubation, non-invasive cells on the upper chambers were discarded using cotton swabs, and invasive cells in the bottom chambers were fixed with 10% methanol and stained with 0.1% crystal violet. Invasive cells were observed and photographed under an inverted microscope (Olympus, Tokyo, Japan). Migration examination was performed similarly, albeit the upper-chambers were non-Matrigel-coated.

### Wound healing assay

Cells were seeded in 24-well plates at a density of 2 × 10^4^ cells/well; 48 h after transfection, a scratch was made using a 200-μl micropipette tip along the central axis of the plate. The cells were gently washed with PBS to wash away the loose cells. Then, cells were treated with 1 μg/ml mitomycin C to inhibit cell proliferation. At 0 h and 24 h of the wounding, the images were recorded, and the relative migratory distance was calculated by comparing the difference in wound width.

### Determination of cytokines by ELISA

The culture medium was collected for ELISA assay using human VEGFA ELISA kits according to the manufacturer's instructions (Santa Cruz, CA, USA). The specific binding optical density was assayed immediately at 450 nm with a spectrophotometer (Bio-Rad Laboratories, Philadelphia, PA, USA).

### HUVECs tubule formation

The knockdown or overexpression of mPRα was generated in lung adenocarcinoma cell line A549; 48 h after transfection, the culture medium was collected as conditioned medium (CM) and used for HUVEC culture. HUVECs were seeded in Matrigel-coated 96-well plates at a density of 1 × 10^4^ cells/well and cultured with the serum-free medium for 6 h. Then, the cells were cultured with different CM as described above for 6 h. Branch numbers or tube lengths were calculated from images in the ImageJ software.

### cAMP levels in culture medium

The levels of cAMP were determined with a DetectX® Cyclic AMP (cAMP) Direct Immunoassay kit (K019-H1; Arbor Assays, Ann Arbor, MI, USA) according to the manufacturer instructions.

### Establishment of Xenograft tumor model in nude mice

A549 cells (1 × 10^7^ cells) were infected with mPRα knockdown or overexpression lentivirus (obtained from GeneChem, Shanghai) and subcutaneously injected to the left flank of BALB/c nude mice (Hunan SJA Laboratory Animal Co., LTD, Changsha, China). Tumor volume was monitored once every three days for 21 days. On day 21, all animals were sacrificed, and the tumor tissues were collected for weight, HE staining, IHC staining, and immunoblotting analysis. All animal procedures gained the approval of Xiangya Hospital of Central South University Ethics Committee.

### Data processing and statistical analysis

All data were processed using GraphPad software (San Diego, CA, USA) and expressed as mean ± standard deviation (SD) from three independent experiments. The Shapiro–Wilks test was used to explore whether data are normally distributed and determine a parametric or non-parametric statistical approach. Kruskal–Wallis test was used for non-parametric statistical analysis. Differences between two groups were assessed by Student's *t*-test. Differences among more than two groups were assessed by a one-way ANOVA followed turkey post-hoc test. Data with *P* values < 0.05 were considered significant.

## Results

### mPRα expression and MVD within tissue samples

**Fig. 1:**
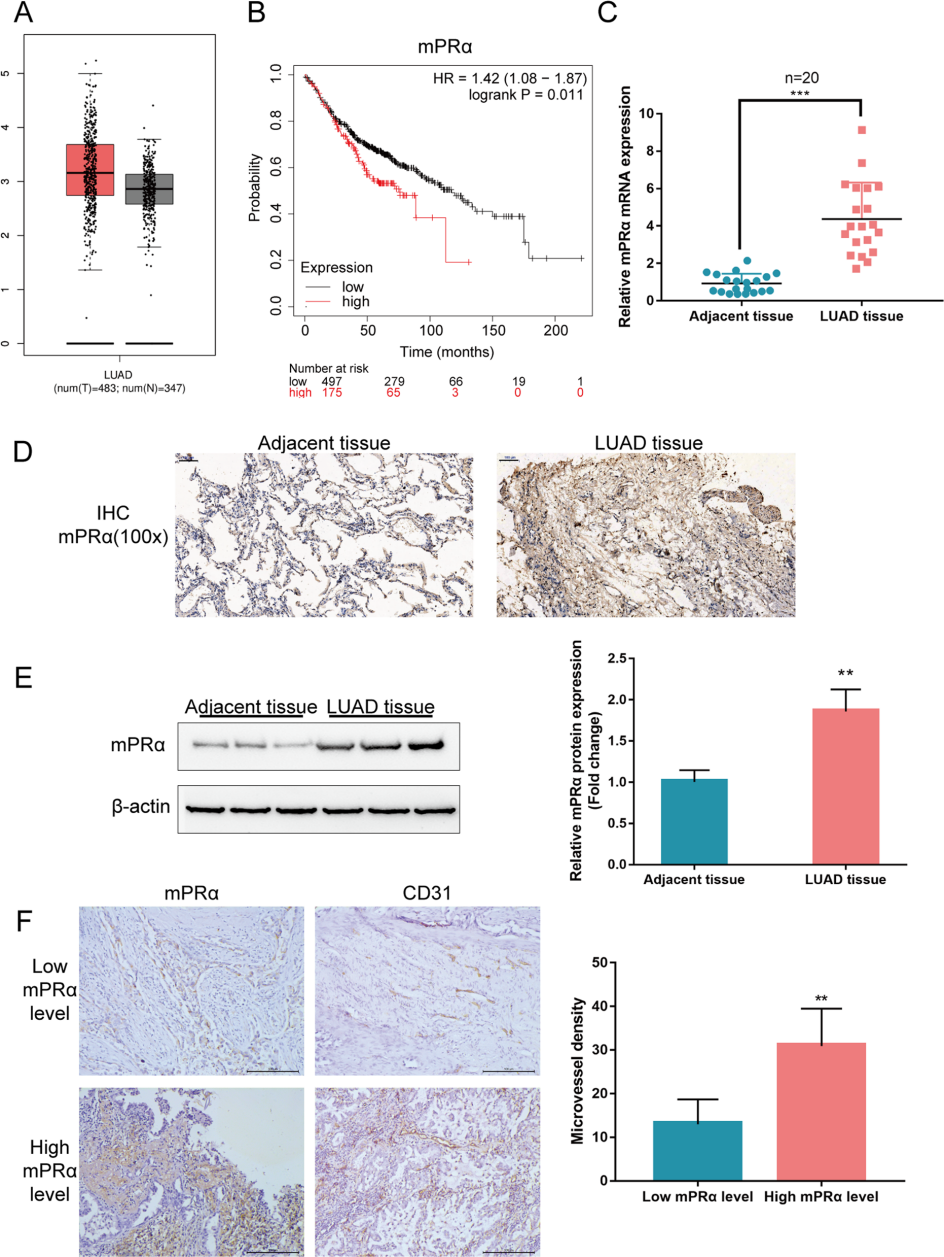
Expression of mPRα in tissue samples. **A** The mRNA expression of mPRα in 483 lung adenocarcinoma (LUAD) tissue samples and 347 noncancerous tissue samples according to TCGA database. **B** Cases from the TCGA-LUAD database were divided into two groups using the best cutoff of mPRα expression. The correlation between mPRα expression and the overall survival in patients with LUAD was analyzed by KMPLOT (https://kmplot.com/analysis/). **C** The mRNA expression of mPRα was determined in 20 paired LUAD and adjacent noncancerous tissue samples using real-time PCR. **D** The protein content and distribution of mPRα were determined in LUAD and adjacent noncancerous tissue samples using immunohistochemical (IHC) staining. **E** The protein content and distribution of mPRα were determined in LUAD and adjacent noncancerous tissue samples using Immunoblotting. **F** The CD31-MVD was determined in LUAD tissues samples with a high or low mPRα level. **P < 0.01, ***P < 0.005

Before investigating the specific effects of mPRα on LUAD carcinogenesis, the study first confirmed the expression level of mPRα in tissue samples. According to TCGA data, mPRα mRNA expression seems to be upregulated within LUAD tissue samples (n = 483) compared to that in noncancerous tissue samples (n = 347), while there is no statistical difference (Fig. 1A). However, higher expression mPRα is associated with a worse overall survival rate in TCGA-LUAD (Fig. 1B). Next, the mRNA expression of mPRα was determined in collected 20 paired LUAD and adjacent noncancerous tissue samples using real-time PCR; consistent with online data, the expression of mPRα showed markedly increased within LUAD tissue samples than that in noncancerous tissue samples (Fig. 1C). As revealed by IHC staining, the protein content of mPRα was higher in LUAD tissue sections than that in noncancerous tissue sections (Fig. 1D). The Immunoblotting assay consistently indicated that the protein level of mPRα was increased in LUAD tissues compared to that in noncancerous tissues (Fig. 1E). As we presumed mPRα might affect angiogenesis in LUAD, MVD in LUAD tissues with different mPRα was calculated, and the result revealed that MVD is significantly higher in LUAD tissues with a higher mPRα level (Fig. 1F). These data suggest that mPRαupregulation in LUAD might have an essential effect on angiogenesis in LUAD tissues.

### mPRα activates cAMP/JAK/STAT3 signaling pathway in LUAD cells

**Fig. 2:**
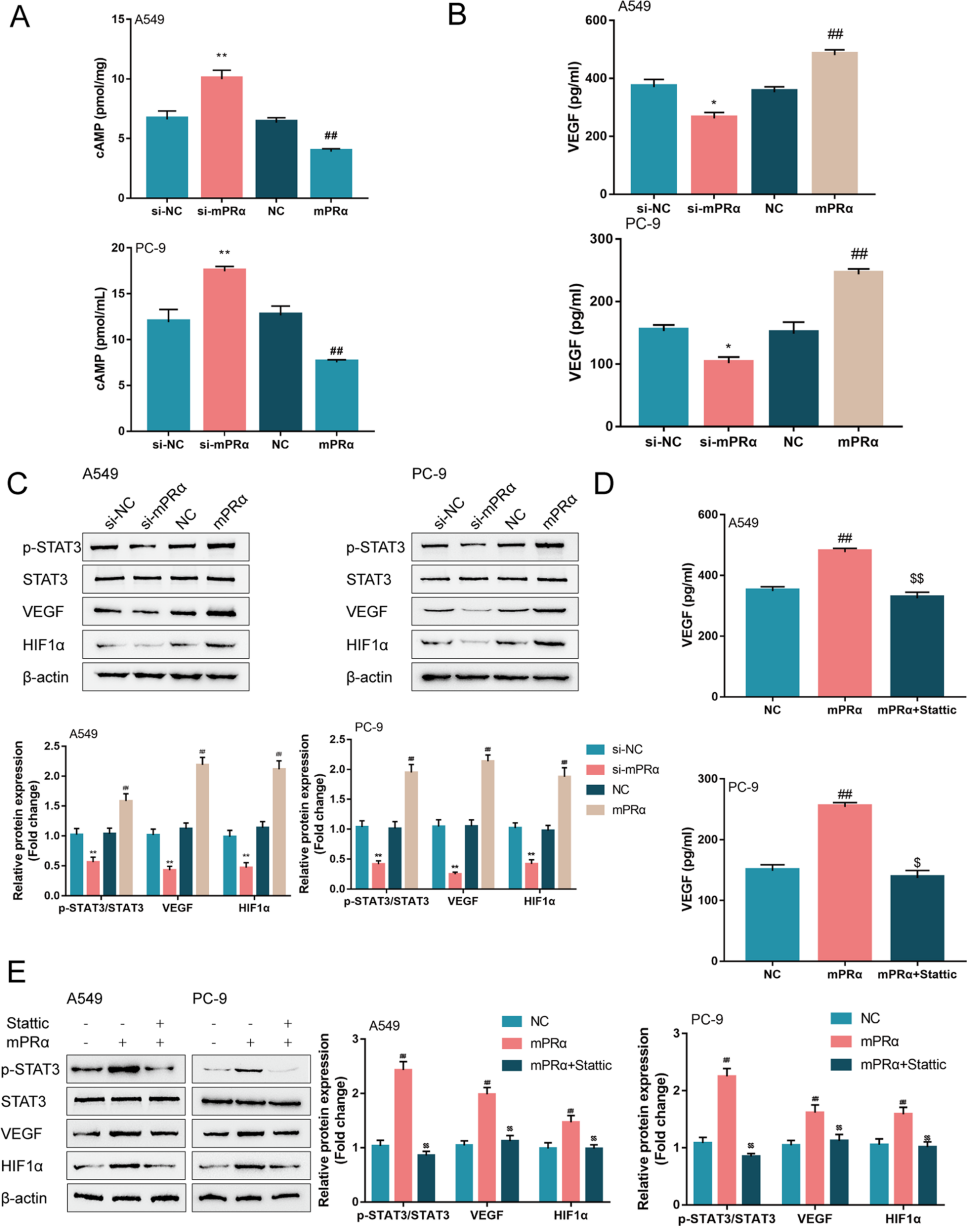
mPRα activates cAMP/JAK/STAT3 signaling pathway in LUAD cells A549 and PC-9 cells were transfected with si-mPRα or mPRα and examined for (**A**) cAMP concentrations using a cAMP Direct Immunoassay kit; **B** VEGF concentrations using ELISA; **C** the protein levels of p-STAT3, STAT3, VEGF, and HIF1α. Next, A549 and PC-9 cells were transfected with mPRα in the presence or absence of STAT3 inhibitor Stattic (5 μM for 24 h) and examined for (**D**) VEGF concentrations using ELISA and **E** the protein levels of p-STAT3, STAT3, VEGF, and HIF1α. *P < 0.05, **P < 0.01, compared to si-NC; #*P* < 0.05, ##*P* < 0.01, ###*P* < 0.005 compared to NC group; $$ *P* < 0.01, compared to mPRα

As we have mentioned, in low oxygen conditions, tumor cells are able to secrete some angiogenesis factors like VEGF into the tumor microenvironment, subsequently enhancing the angiogenesis under hypoxia [18, 35]. Besides, after STAT3 activation, HIF1α-induced VEGF release was increased [21]. Thus, the study generated mPRα knockdown or overexpression in two LUAD cell lines, A549 and PC-9, by transducing siRNA for mPRα (si-mPRα) or mPRα-overexpressing vector (mPRα) and detected the effects of mPRα on cAMP/JAK/STAT3 signaling pathway and the content of HIF1α and VEGF, using real-time PCR (Additional file 1: Fig. S1A) and Immunoblotting (Additional file 1: Fig. S1B). In both cell lines, mPRα knockdown significantly increased the cAMP concentrations, reduced the VEGF concentrations, decreased the ratio of p-STAT3/STAT3, and decreased the protein levels of VEGF and HIF1α (Fig. 2A–C); in contrast, mPRα overexpression exerted opposite effects by decreasing the concentrations of cAMP, increasing the concentrations of VEGF, increasing the ratio of p-STAT3/STAT3, and increasing the protein levels of VEGF and HIF1α (Fig. 2A–C). For investigating whether mPRα exerts its effects through STAT3 signaling, the study transfected A549 and PC-9 cells with mPRα with or without STAT3 inhibitor Stattic, and examined the same indexes. As shown in Fig. 2D, E, mPRα overexpression-induced increases in VEGF concentrations, p-STAT3/STAT3 ratio, and the protein levels of VEGF and HIF1α were significantly decreased by STAT3 inhibitor Stattic. Thus, based on these data, we speculate that mPRα might activate the STAT3 signaling, subsequently promoting HIF1α-induced VEGF release and angiogenesis under hypoxia.

### mPRα promoted LUAD cells invasion, migration, and tumor growth

**Fig. 3:**
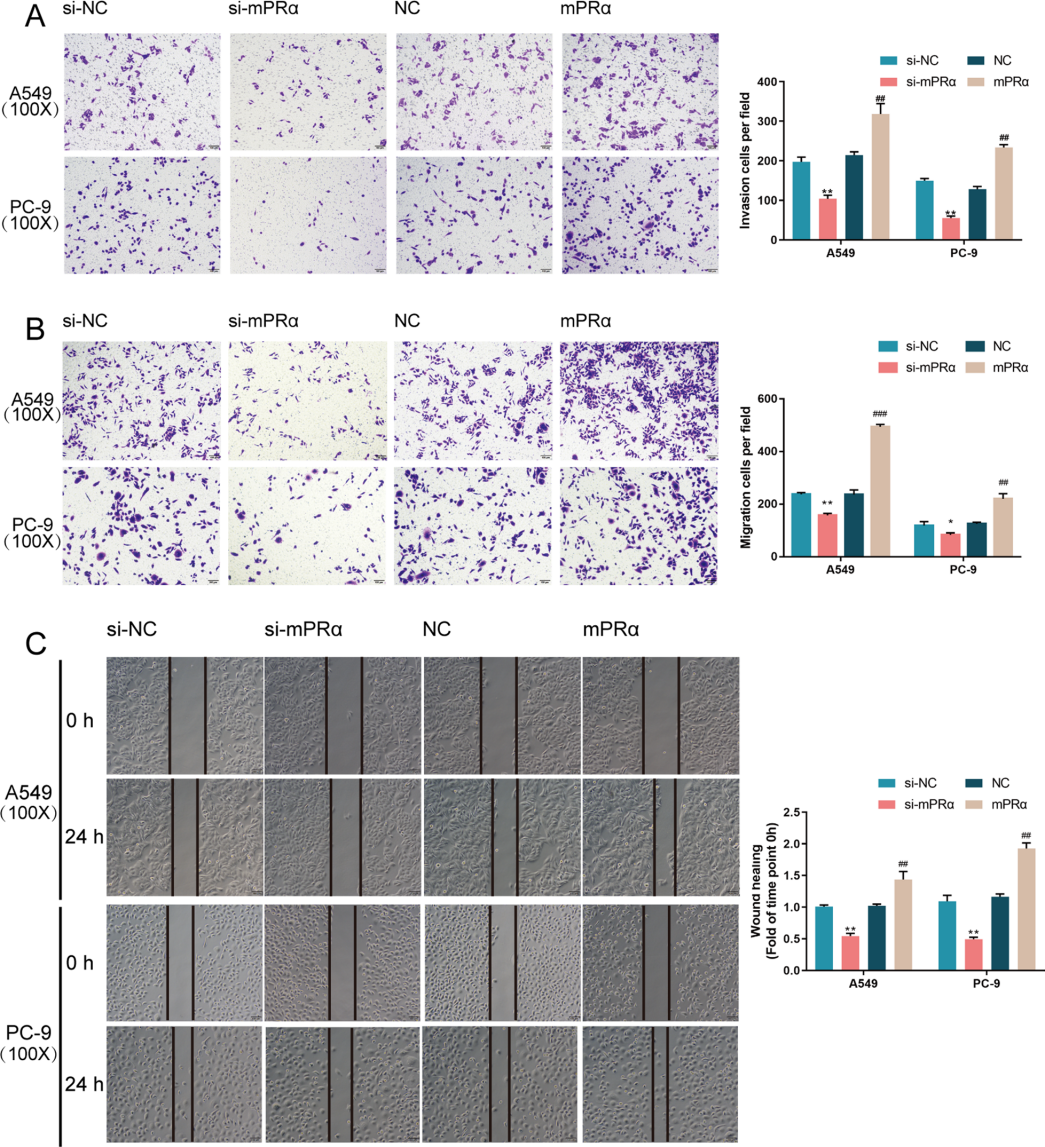
Specific effects of mPRα on LUAD cell migration and invasion Next, A549 and PC-9 cells were transfected with si-mPRα or mPRα and examined for **A**, **B** cell migration and invasion by Transwell assays and **C** cell migration by wound healing assay. *P < 0.05, **P < 0.01, compared to si-NC; #*P* < 0.05, ##*P* < 0.01, ###*P* < 0.005 compared to NC group

After confirming VEGF upregulated by mPRα in LUAD tissues, we evaluated the capacity of cells to migrate and invade, which could be promoted by VEGF. Based on both Transwell and Wound healing assays, mPRα knockdown significantly inhibited, while mPRα overexpression promoted the cell migration of both A549 and PC-9 cell lines (Fig. 3A and C). Similarly, mPRα knockdown significantly inhibited, while mPRα overexpression promoted the cell invasion of both A549 and PC-9 cell lines (Fig. 3B).

**Fig. 4:**
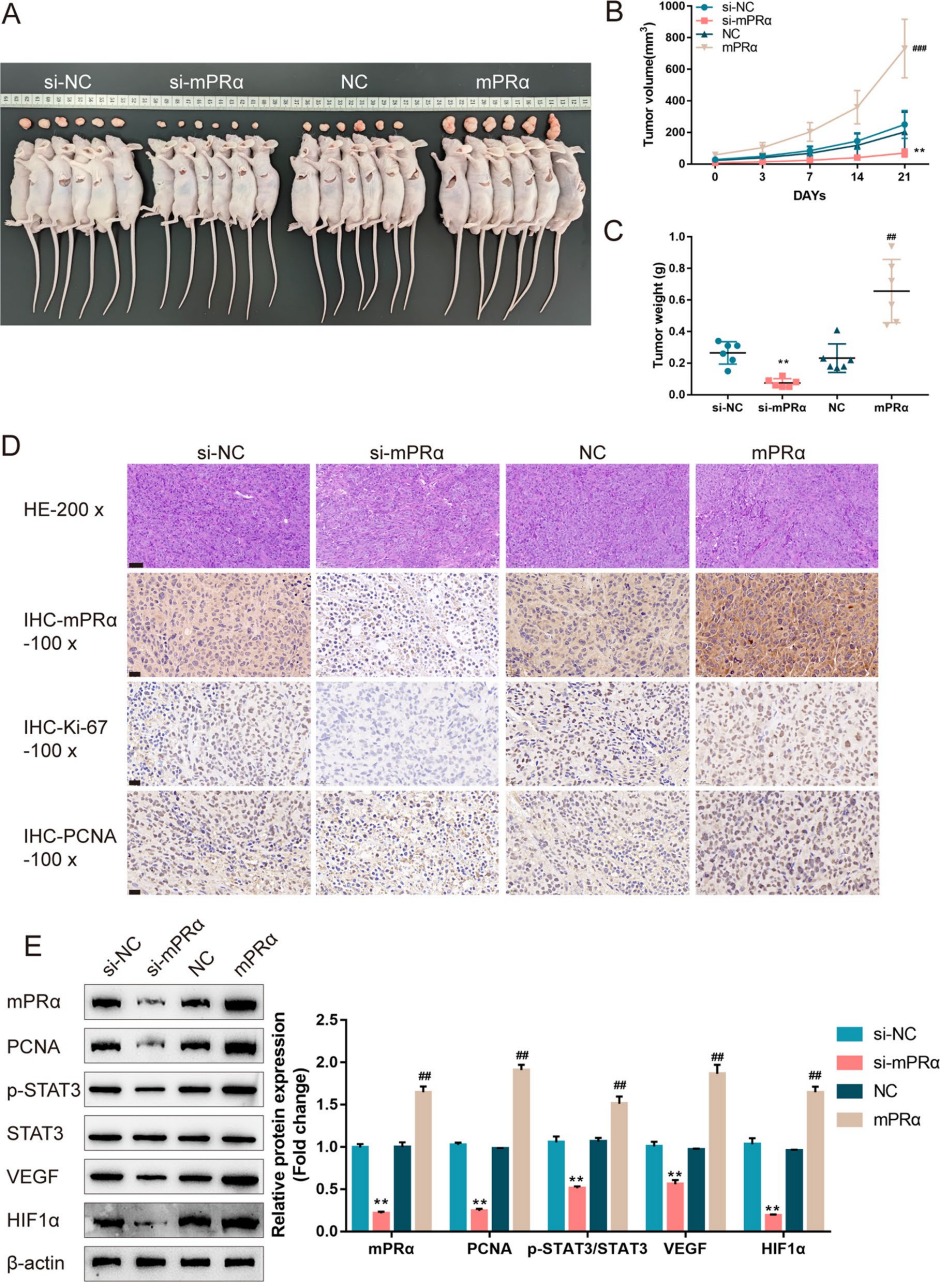
mPRα promoted LUAD cell growth in the xenograft model. **A–C** xenograft mouse tumor model was established in BALB/C nude mice accordingly. MPRα knockdown or overexpression lentivirus infected A549 cells were injected under the skin of the left flank of nude mice. The tumor volume **B** was measured every 3 days for 21 days; on day 21, mice were sacrificed, and the tumor weight was measured (**C**); **D** the protein levels of mPRα, ki67, and PCNA in tumor tissues were examined using IHC staining. **E** The protein levels of mPRα, PCNA, p-STAT3, STAT3, VEGF, and HIF1α in tumor tissues were examined using Immunoblotting. **P < 0.01, compared to si-NC. ## *P* < 0.01, ### *P* < 0.005, compared to NC

Next, for investigating the in vivo effects of mPRα on LUAD growth, A549 cells were infected with lentivirus containing mPRα shRNA or overexpression fragment and used for xenograft model establishment. As shown in Fig. 4A–C, mPRα knockdown significantly repressed tumor growth. In contrast, mPRα overexpression enhanced tumor growth. Correspondingly, mPRα, ki67, PCNA, p-STAT3, VEGF and HIF1α proteins also decreased within mPRα knockdown tumors and increased within mPRα overexpression tumors (Fig. 4D, E). These data indicate that mPRα enhances the aggressiveness of LUAD cells.

### mPRα knockdown decreases hypoxia-induced HIF1α/VEGF content and LUAD cell aggressiveness

**Fig. 5:**
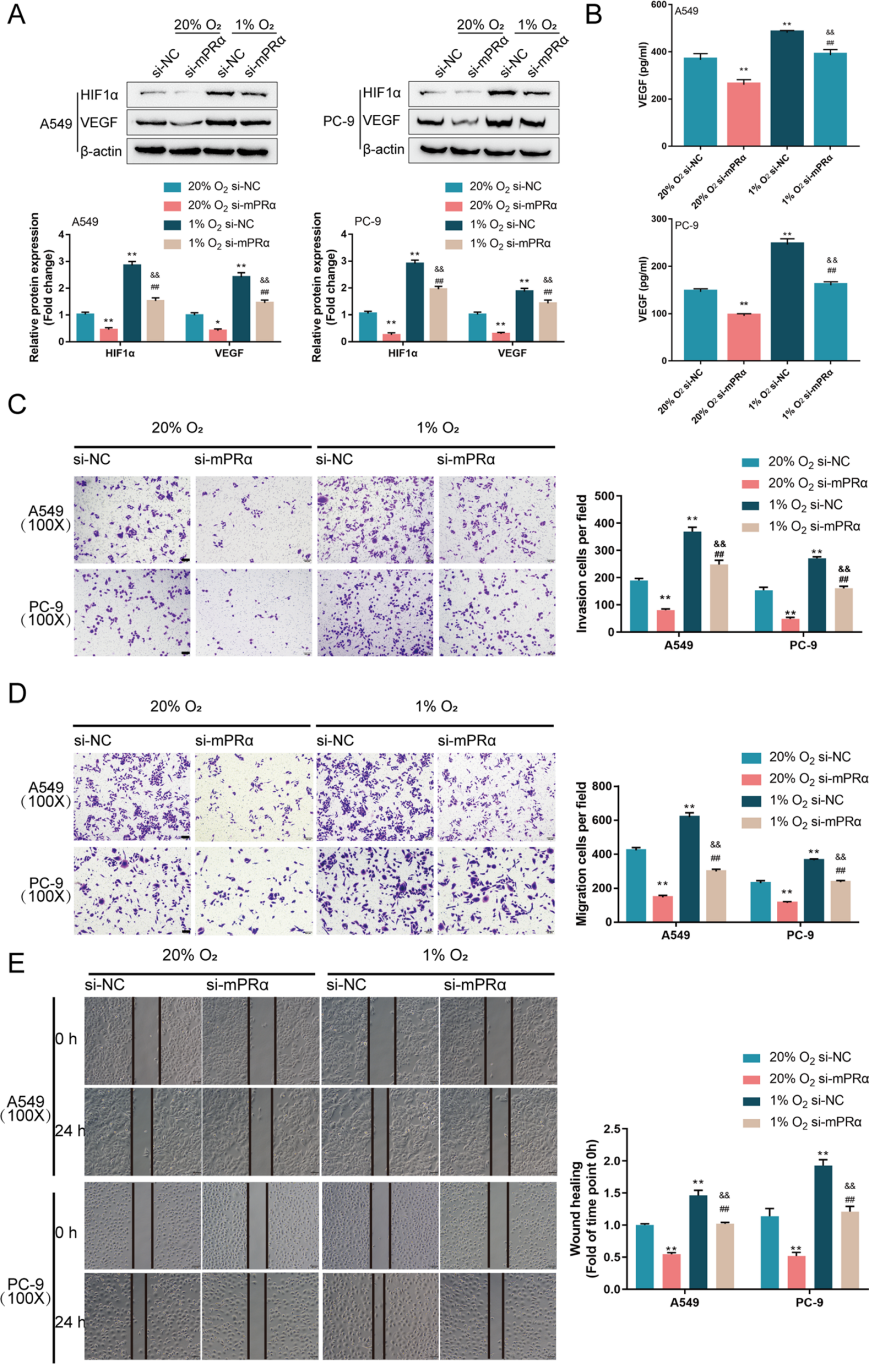
mPRα knockdown decreases hypoxia-induced HIF1α/VEGF content and LUAD cell aggressiveness A549 and PC-9 cells were transfected with si-mPRα or si-NC, exposed to hypoxia (1% O_2_) or normoxia (20% O_2_), and examined for **A** the protein levels of HIF1α and VEGF by Immunoblotting; **B** VEGF concentrations by ELISA; **C**, **D** cell migration and invasion by Transwell assays; **E** cell migration by wound healing assay. *P < 0.05, **P < 0.01, compared to 20% O_2_ si-NC; ##*P* < 0.01, compared to 20% O_2_ + si-mPRα; &&*P* < 0.01, compared to 1% O_2_ si-NC group

The specific role of mPRα in HIF1α-induced VEGF release and LUAD cell aggressiveness was investigated under hypoxia. A549 and PC-9 cells were transfected with si-mPRα or si-NC (negative control), exposed to hypoxia (1% O_2_) or normoxia (20% O_2_), and examined for related indexes. Consistent with previous studies, hypoxia exposure significantly increased HIF1α and VEGF protein contents and VEGF concentrations in culture medium compared to those under normoxic conditions (Fig. 5A, B). After knocking down mPRα, hypoxia-induced increases in HIF1α and VEGF levels were partially attenuated (Fig. 5A, B). As for the LUAD cell aggressiveness, hypoxia increased the capacity of these two cell lines to migrate and to invade (Fig. 5C–E), while mPRα knockdown inhibited hypoxia-induced LUAD cell migration and invasion (Fig. 5C–E).

### mPRα enhances the tube formation and migration capacity of HUVECs

Since mPRα knockdown inhibited hypoxia-induced HIF1α and VEGF levels, as well as LUAD cell aggressiveness, we speculate that mPRα might also play a role in HUVEC tube formation by affecting HIF1α-induced VEGF release through STAT3 signaling under hypoxia.

**Fig. 6:**
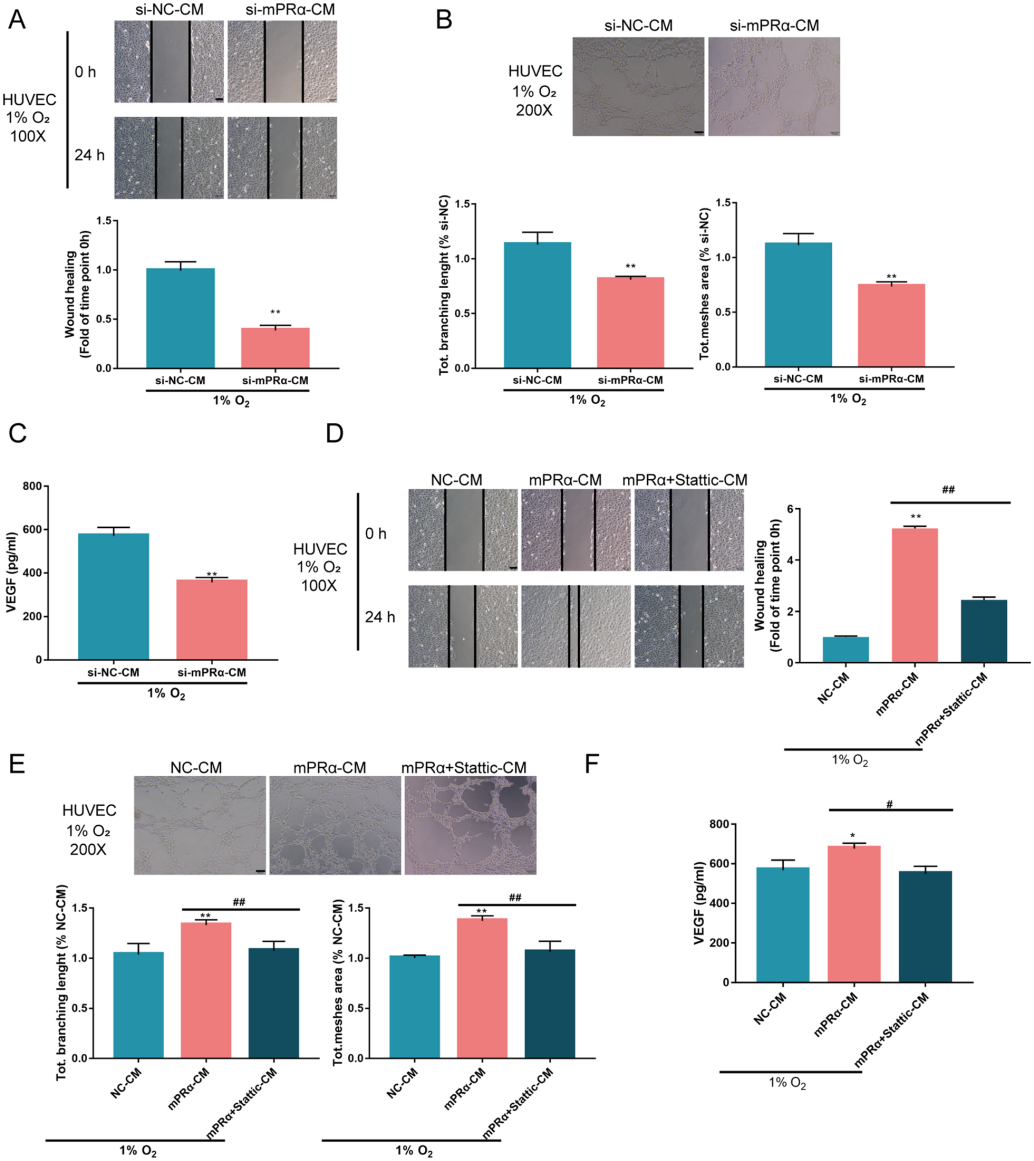
mPRα enhances HUVEC tube formation and migration under hypoxia through HIF1α-induced VEGF release, and STAT3 signaling A549 cells were transfected with si-mPRα or si-NC (negative control). The culture medium was collected for HUVEC culture (marked as si-mPRα-CM/si-NC-CM). HUVECs were cultured in different conditioned mediums under 1% O_2_ and examined for **A** cell migration by wound healing assay, **B** tube formation by Tube formation assay, and **C** VEGF content in the culture medium by ELISA. Next, A549 cells were transfected with NC (negative control) or mPRα-overexpressing vector, and the culture medium was collected for HUVEC culture (marked as NC-CM or mPRα-CM). HUVECs were cultured in different conditioned mediums in the presence or absence of STAT3 inhibitor Stattic under hypoxia (1% O_2_) and examined for **D** cell migration by wound healing assay, **E** tube formation by Tube formation assay, and **F** VEGF content in the culture medium by ELISA. *P < 0.05, **P < 0.01, compared to NC or si-NC-CM; #*P* < 0.05, ##*P* < 0.01 compared to mPRα-CM

We transfected A549 cells with si-mPRα/si-NC and collected the culture medium for HUVEC culture (marked as si-mPRα-CM/si-NC-CM in Fig. 5). We cultured HUVECs in these CMs under hypoxia and examined HUVEC migratory and tube formation capacities. As shown in Fig. 6A, B, si-mPRα-CM culture significantly inhibited HUVEC migration and tube formation under hypoxia. Moreover, under hypoxia, VEGF release into the culture medium was reduced by si-mPRα-CM (Fig. 6C). These data indicate that mPRα affects HIF1α-induced VEGF release into the tumor microenvironment, modulating hypoxia-induced angiogenesis in LUAD.

Next, we transfected A549 cells with mPRα/NC and collected the culture medium for HUVEC culture (marked as NC-CM/mPRα-CM in Fig. 6). HUVECs were cultured in different CMs, exposed to hypoxia (1% O_2_) in the presence or absence of STAT3 inhibitor Stattic, and examined for HUVEC migratory and tube formation capacities. Figure 6D, E showed that hypoxia significantly induced HUVEC migration and tube formation, and mPRα-CM culture further enhanced the effects of hypoxia; however, treatment with Stattic significantly attenuated the inducible effects of hypoxia and mPRα-CM on HUVEC migration and tube formation. Moreover, hypoxia-induced VEGF release was further enhanced by mPRα-CM culture but reduced by Stattic treatment (Fig. 6F). These data indicate that STAT3 signaling is involved in mPRα functions on LUAD cells and HUVECs.

## Discussion

Herein, mPRα deregulation was observed within LUAD tissue samples. mPRα knockdown in A549 and PC-9 cells significantly inhibited STAT3 phosphorylation, as well as HIF1α and VEGF protein levels, suppressing the capacity of LUAD cells to migrate and to invade. Under the hypoxic condition, mPRα knockdown significantly inhibited hypoxia-induced increases in HIF1α and VEGF levels, as well as LUAD cell migration and invasion. Moreover, CM derived from mPRα knockdown A549 cells, namely si-mPRα-CM, significantly inhibited HUVEC migration and tube formation under hypoxia.

mPRα is frequently deregulated in many cancers. Within LUAD, online data from TCGA indicated an abnormal upregulation of mPRα expression in LUAD tissue samples. mPRα mRNA expression and protein levels were also increased in LUAD subjects. However, regarding the specific role of mPRα in carcinogenesis, opposite results have been reported, depending on the cancer type. In glioblastoma, treatment with the specific mPRα agonist Org OD 02–0 induced an increase in U87 and U251 cell count by promoting the capacity of cells to proliferate; in addition, this treatment significantly promoted the capacity of U87 and U251 cells to migrate and invade [15]. As for breast cancer, Org OD 02–0 treatment dramatically inhibited the death and apoptosis of cells under serum deficiency stimulation [30]. Another group also regarded mPRα as a key marker of impaired prognosis for invasive breast cancer, and mPRα enhanced MMP-9 expression in the process of spreading to regional lymph nodes via the PI3K/Akt pathway [12]. Reportedly, mPRα could promote breast cancer resistance protein (BCRP) expression via the PI3K/Akt/mTOR signaling pathway, contributing to metastasis and drug resistance [17]. Herein, we observed consistent results that mPRα are positively correlated to CD31-MVD in LUAD tissues, indicating the carcinogenic effect of mPRα on LUAD.

As for the involved signaling pathway, herein, we next validated the roles of mPRα in cAMP/JAK/STAT3 signaling and related HIF1α-induced VEGF release. Consistent with previous studies, mPRα overexpression significantly decreased the cAMP concentrations and increased VEGF concentrations, increased the phosphorylation of STAT3, and increased the protein levels of HIF1α and VEGF. Moreover, after knocking down mPRα in LUAD cells, the cell migration, invasion, and in vivo tumor growth were significantly inhibited, which could be explained by the changes in VEGF expression. However, mPRα has been reported to mediate the suppressive functions of PR [36–38], even in A549 cells [39]. These seemingly contradictory findings suggest that mPRα might function in different ways in LUAD, with the presence or absence of PR, which needs further investigation in our future study. Moreover, in mPRα-overexpressing LUAD cells, the treatment with STAT3 inhibitor Stattic significantly attenuated the inducible effects of mPRα overexpression on STAT3 phosphorylation, HIF1α, and VEGF protein contents, as well as VEGF concentrations, suggesting that mPRα promotes HIF1α-induced VEGF release through cAMP/JAK/STAT3 signaling. Receptor-related JAKs are well-known mediators of STAT3 phosphorylation for tumor development and inflammatory response [40, 41]. Herein, mPRα overexpression significantly promoted STAT3 phosphorylation, further confirming the oncogenic role of mPRα in LUAD. STAT3 is also tightly related to VEGF in cancer progression [42, 43]. STAT3 is a transcription factor that enhances the progression of urothelial cells from carcinoma in situ to invasive bladder cancer and regulates the angiogenesis of RCC (renal cell carcinoma) through upregulating HIF1α and VEGF expression [44]. Moreover, under hypoxia, phosphorylated STAT3 enhances HIF1α stability to increase HIF1α-mediated VEGF secretion, promoting angiogenesis in renal carcinoma and liver cancer [45, 46]. Herein, mPRα overexpression increased HIF1α and VEGF protein levels and VEGF concentrations in culture medium, suggesting the potential role of mPRα in hypoxia-induced angiogenesis in LUAD.

Tumor micro-environment hypoxia shows to be closely related to tumor development [47]. Oxygen homeostasis is directly regulated via HIF1α; following a shift to a low-oxygen environment, HIF1α is stabilized and subsequently translocated into the nucleus. Nuclear-translocated HIF1α leads to the expression of some genes, including VEGF [48]. VEGF is a mitogen for vascular endothelial cells and several other cell types, eliciting a pronounced angiogenic response [49]. In LUAD, miR-204 targets JAK2 to inhibit JAK/STAT3 signaling, subsequently decreasing the mRNA and protein expression of HIF1α and VEGF within LUAD A549 cells; conditioned medium from miR-204 overexpressed A549 cells obviously decreased the cumulative tube length and migratory ability of HUVECs [50]. Herein, hypoxia significantly increased HIF1α and VEGF protein contents and promoted LUAD cell migration and invasion, which were all remarkably reversed by mPRα knockdown. Moreover, hypoxia also enhanced the migratory ability and tube formation capacity of HUVECs, which showed to be reversed by the incubation with the conditioned medium derived from mPRα knocked-down A549 cells; these findings indicate that mPRα knockdown could reduce HIF1α-induced VEGF secretion into tumor microenvironment, therefore impairing hypoxia-induced angiogenesis in HUVECs. Furthermore, conditioned medium derived from mPRα-overexpressing A549 cells enhanced the effects of hypoxia on HUVEC angiogenesis and VEGF content in the culture medium, which were reversed by STAT3 inhibitor Stattic, indicating that STAT3 signaling is involved in mPRα effects on LUAD cells and HUVECs.

In conclusion, we demonstrated that in LUAD cells, highly expressed mPRα enhances the activation of cAMP/JAK/STAT3 signaling and increases HIF1α-induced VEGF secretion into the tumor microenvironment, promoting HUVEC migration and tube formation under hypoxia.

## Supplementary Information

**Additional file 1: Table S1.** the sequence of primers, siRNA and plasmid construction.

**Additional file 2: Figure S1.** mPRα knockdown or overexpression generated in two LUAD cell lines, A549 and PC-9, by the transfection of siRNA for mPRα (si-mPRα) or mPRα-overexpressing vector (mPRα), as confirmed by (A) real-time PCR and (B) Immunoblotting. **P<0.01, compared to si-NC. ## P<0.01, compared to NC.

## Data Availability

All datasets generated for this study are included in the article.
