# Supplementary figures and images for: Membrane progesterone receptor α (mPRα) enhances hypoxia-induced vascular endothelial growth factor secretion and angiogenesis in lung adenocarcinoma through STAT3 signaling
Source: J Transl Med. 2022 Feb 5;20:72. doi: 10.1186/s12967-022-03270-5 (PMC8817580; doi:10.1186/s12967-022-03270-5)

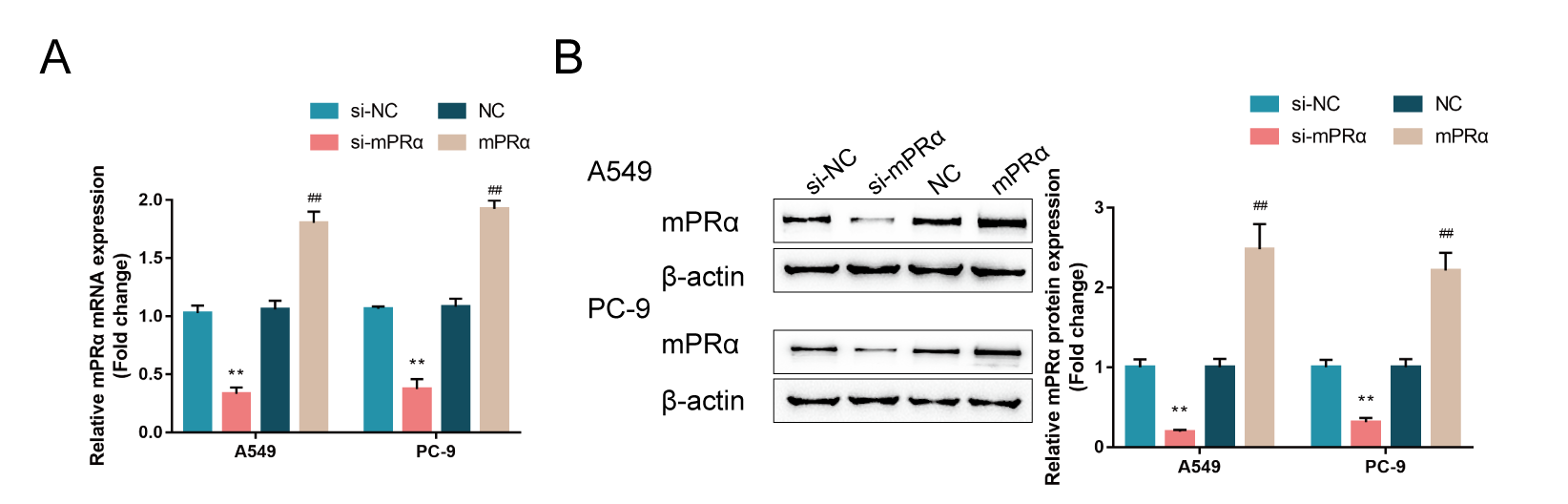

Supplement: Supplementary file 1 — Additional file 1: Table S1. the sequence of primers, siRNA and plasmid construction. [file 12967_2022_3270_MOESM1_ESM.tif]
