# Supplementary material for: Membrane progesterone receptor α (mPRα) enhances hypoxia-induced vascular endothelial growth factor secretion and angiogenesis in lung adenocarcinoma through STAT3 signaling
Source: J Transl Med. 2022 Feb 5;20:72. doi: 10.1186/s12967-022-03270-5 (PMC8817580; doi:10.1186/s12967-022-03270-5)
Supplement: Supplementary file 2 — Additional file 2: Figure S1. mPRα knockdown or overexpression generated in two LUAD cell lines, A549 and PC-9, by the transfection of siRNA for mPRα (si-mPRα) or mPRα-overexpressing vector (mPRα), as confirmed by (A) real-time PCR and (B) Immunoblotting. **P<0.01, compared to si-NC. ## P<0.01, compared to NC. [file 12967_2022_3270_MOESM2_ESM.docx]

Table S1 the sequence of primers, siRNA and plasmid construction

|  | Forward 5’-3’ | Reverse 5’-3’ |
| --- | --- | --- |
| RT-PCR  mPRα | CTCTTTCACCTACCTCTCCTTCA | AAGCTGTAATGCCAGAACTCAG |
| RT-PCR  β-actin | TTCCAGCCTTCCTTCCTGGG | TTGCGCTCAGGAGGAGCAAT |
| Si-NC | UUCUCCGAACGUGUCACGUTT | ACGUGACACGUUCGGAGAATT |
| Si- mPRα | GCUUUGAUAUCAGAAUGAATT | UUCAUUCUGAUAUCAAAGCTT |
| PcDNA3.1 mPRα overexpression | ctagcgtttaaacttaagcttATGGCCATGGCCCAGAAA | tgctggatatctgcagaattcTCACTTGGTCTTCTGATCAAGTTTG |
| Lentivirus  mPRα knockdown | GATCCGCCTGGATGCAATAAAGTACTCTCGAGAGTACTTTATTGCATCCAGGCTTTTTG | AATTCAAAAAGCCTGGATGCAATAAAGTACTCTCGAGAGTACTTTATTGCATCCAGGCG- |
| Lentivirus  mPRα overexpression (plvx-puro) | ctaccggactcagatctcgagATGGCCATGGCCCAGAAA | gtaccgtcgactgcagaattcTCACTTGGTCTTCTGATCAAGTTTG |
| Lentivirus  Knockdown NC | GATCCGCAGATGAAGGCACGGTCACGCTCGAGGCAGATGAAGGCACGGTCACGTTTTTG | AATTCAAAAAGCAGATGAAGGCACGGTCACGCTCGAGGCAGATGAAGGCACGGTCACGG |
